# The Real-World Effectiveness, Persistence, Adherence, and Safety of Janus Kinase Inhibitor Baricitinib in Rheumatoid Arthritis: A Long-Term Study

**DOI:** 10.3390/jcm13092517

**Published:** 2024-04-25

**Authors:** Alberto Calvo-Garcia, Esther Ramírez Herráiz, Irene María Llorente Cubas, Blanca Varas De Dios, Juana Benedí González, Alberto Morell Baladrón, Rosario García-Vicuña

**Affiliations:** 1 Pharmacy Service, Hospital Universitario La Princesa, IIS-Princesa, 28006 Madrid, Spain; alberto.calvo@salud.madrid.org (A.C.-G.); eramirezh@salud.madrid.org (E.R.H.); alberto.morell@salud.madrid.org (A.M.B.); 2 Rheumatology Service, Hospital Universitario La Princesa, IIS-Princesa, 28006 Madrid, Spain; irenemaria.llorente@salud.madrid.org; 3 Rheumatology Service, Hospital Universitario Santa Cristina, 28006 Madrid, Spain; blanca.varas@salud.madrid.org; 4 Pharmacology, Pharmacognosy and Botany Department, Pharmacy Faculty, Complutense University of Madrid, 28040 Madrid, Spain; jbenedi@farm.ucm.es; 5 Department of Medicine, Faculty of Medicine, Autonomous University of Madrid, 28049 Madrid, Spain

**Keywords:** JAK-inhibitor, baricitinib, rheumatoid arthritis, real-word data, persistence, adherence, safety, unmet needs

## Abstract

**Background/Aim**: Baricitinib (BAR) is the first oral selective Janus kinase inhibitor approved in Europe for rheumatoid arthritis (RA). Real-world data are still needed to clarify its long-term benefits/risk profile. This study aimed to evaluate the effectiveness, persistence, adherence, and safety of BAR in a real-world setting. **Methods**: An ambispective study was conducted between October 2017 and December 2021 in RA patients starting BAR. The effectiveness was evaluated, assessing changes from the baseline of the Disease Activity Score using 28-joint counts-C reactive protein (DAS28CRP), and the achievement of low disease activity/remission. Drug persistence was evaluated using Kaplan–Meier analysis. Adherence was estimated using the medication possession ratio (MPR) and the 5-item Compliance Questionnaire for Rheumatology. Safety was assessed determining global incidence proportion and adverse event adjusted incidence rates. **Results**: In total, 61/64 recruited patients were finally analyzed, 83.6% were female, 78.7% were seropositive, the mean age was 58.1 (15.4) years, and the disease duration was 13.9 (8.3) years. A total of 32.8% of patients were naïve to biologics and 16.4% received BAR as monotherapy. The median exposure to BAR was 12.4 (6.6–31.2) months (range 3.1–51.4). A significant change in DAS28CRP was observed after treatment (difference −1.2, *p* = 0.000). 70.5% and 60.7% of patients achieved low disease activity or remission, respectively, and 50.8% (31/61) remained on BAR throughout the follow-up, with a median persistence of 31.2 (9.3–53.1) months. The average MPR was 0.96 (0.08) and all patients exhibited “good adherence” according to the questionnaire. In total, 21.3% of patients discontinued baricitinib due to toxicity. **Conclusions**: In our real-world practice, BAR demonstrated effectiveness, large persistence, high adherence to treatment, and an acceptable safety profile.

## 1. Introduction

Rheumatoid arthritis (RA) is a chronic autoimmune disease, typically characterized by polyarticular joint inflammation, with potential extra-articular involvement and frequent comorbidities. The persistent inflammation produces a decrease in the patients’ functional capacity and in their quality of life [1].

The physiopathology of RA includes chronic synovial membrane inflammation with the subsequent destruction of the joint cartilage and bone [2]. The current pathogenic model proposes autoimmunity as the main disease trigger in genetically predisposed individuals, resulting in the early presence of circulating auto-antibodies to environmental-induced neoepitopes (anti-citrullinated protein antibodies [ACPAs] and antibodies against immunoglobulin G, such as the rheumatoid factor [RF]) [2].

The main goal of RA treatment is to achieve remission or, at least, low disease activity (LDA) through a “treat to target” strategy [1]. Briefly, disease activity target goals are defined at disease onset, and this activity is tightly monitored, aiming to adjust treatment until the predefined goals are achieved. Disease activity can be assessed using composite indices, such as the Disease Activity Score, using 28-joint counts (DAS28), the Clinical Disease Activity Index (CDAI), and the Simplified Disease Activity Index (SDAI) [1].

Current treatment guidelines for RA recommend conventional synthetic disease-modifying anti-rheumatic drugs (csDMARDs) as the first step in treatment, and when the treatment goals are not met, they endorse adding or switching to either a biologic DMARD (bDMARD), or a targeted synthetic DMARD (tsDMARD) [3]. tsDMARDs inhibit intracellular signaling pathways, specifically the Janus kinase/signal transducer and the activator of the transcription (JAK-STAT) pathway. Baricitinib (BAR), which predominantly inhibits JAK1 and JAK2 isoenzymes, was one of the first JAK inhibitors (JAKi) available for RA treatment [4].

In randomized clinical trials (RCT), BAR demonstrated efficacy in patients naïve to bDMARDs and in those with an inadequate response to csDMARDs or Tumor Necrosis Factor inhibitors (TNFi) [5,6,7]. Furthermore, BAR, in combination with methotrexate (MTX), achieved better results in some early disease activity outcomes than the combination of MTX with adalimumab (ADA) [8].

The increasing incorporation of JAKi into RA therapy, as well as emerging safety issues regarding tofacitinib [9] and even BAR [10], warrant more real-world data (RWD) on effectiveness, safety, and persistence of BAR, in order to consolidate this drug as one of the alternatives for RA treatment. In addition, since the complexity and chronicity of anti-rheumatic treatment may influence adherence, monitoring adherence is mandatory to identify adherence problems and tailor the interventions to solve them [11]. The lack of medication compliance may lead to early treatment failure and the switch to more intensive treatments. Therefore, this study was designed to evaluate the effectiveness, persistence, adherence, and safety of BAR in a real-world setting.

## 2. Materials and Methods

### 2.1. Study Design and Population

**Figure 1:**
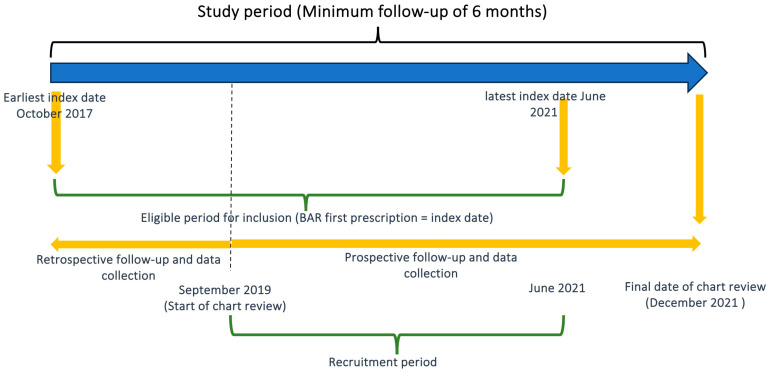
Overall study design. The index date is defined as the first baricitinib prescription date. The follow-up period is defined as the interval from the index date until the date of the final chart review, or any event resulting in the early end of follow-up, treatment withdrawal, or loss to follow-up, whichever comes first.

This was a longitudinal ambispective chart review conducted between October 2017 and December 2021 in La Princesa University Hospital in Madrid, Spain (See Figure 1 for study design). Patients eligible for inclusion were aged ≥18 years, diagnosed with RA according to the American College of Rheumatology (ACR)/European League against Rheumatism (EULAR) 2010 classification criteria [12], and had initiated BAR treatment between October 2017 and June 2021. All consecutive patients whose BAR prescription started within in this inclusion period were recruited for the study. The index date was defined as the first BAR prescription date, and the follow-up was defined as the period between the index date and death, loss to follow-up, or last chart review data, whichever came first. The patient recruitment period lasted from September 2019 to June 2021 and the chart review covered data collection from October 2017 to December 2021. For patients who initiated BAR before September 2019, data were collected retrospectively until that date; subsequently, these patients were followed together with the rest of the patients until December 2021, to ensure that patients initiating BAR in June 2021 had a minimum follow-up of 6 months. The population sample size was calculated based on data from our hospital pharmacy electronic prescription records, where an average of 75 RA patients per year were eligible to start on or switch to bDMARDs or JAK inhibitors (JAKi). Employing a confidence level of 95% and a margin of error of 5%, along with an expected loss of 10–15% during the follow-up period, we determined that a sample size of 64–67 would adequately represent our population. 

The indication for BAR did not include the recent recommendations from The Pharmacovigilance Risk Assessment Committee (PRAC), endorsed by the European Medicines Agency, to minimize the risk of serious side effects of JAKi, because they were published after the end of the observation period [13].

### 2.2. Outcomes

The pre-defined primary endpoints for effectiveness were changes from the baseline in DAS28-C reactive protein (CRP), and rates of low disease activity (LDA) (2.6 < DAS28-erytrocyte sedimentation rate [ESR] ≤ 3.2), and disease remission (DAS28CRP < 2.6) [14] at months 6, 12, 24, and at the end of follow-up. As the secondary endpoint, rates of EULAR response at the end of the follow-up were also assessed. The DAS28CRP score was chosen to evaluate effectiveness, based on EULAR/ACR recommendations for defining RA activity in studies with patients and its widespread use in clinical practice [15].

The primary endpoints for treatment persistence were the number of days on BAR treatment until discontinuation or the end of follow-up period (compiled from electronic prescribing and dispensing records) [16] and the rate of patients who maintained BAR at 6, 12, 24, 36, and 48 months and at the end of follow-up period. In addition to overall persistence, the median persistence was differentially assessed in patients who discontinued treatment due to a loss of effectiveness or to toxicity. Adherence was calculated using two methods. The first method was the assessment of the medication possession ratio (MPR), defined as the sum of the days’ supply for all fills of the drug in a given time period divided by the number of days in this period (compiled from electronic prescribing and dispensing records). Patients were considered adherent when their MPR was ≥0.8 [17]. The second method was the 5-item Compliance Questionnaire for Rheumatology (CQR5), a simplified and validated Spanish version of the 19-item CQR [18,19,20]. This questionnaire was only applied to patients with a prospective follow-up.

To evaluate safety, the global incidence proportion (IP) and adjusted incidence rate (IR) per 100 patient years (PY) of adverse events (AEs) were calculated. Deviations in laboratory values were defined according to the specifications in the BAR summary of product characteristics [21]. AEs were classified according to the Common Terminology Criteria for Adverse Events (CTCAE) Version 5.0 [22]. 

### 2.3. Statistical Analysis

Summary statistics are expressed as means (standard deviation) or medians (25–75 interquartile range [IQR]) for quantitative variables, or n (percentage) for qualitative variables. Paired analyses of DAS28CRP and laboratory values were performed using the Wilcoxon signed-rank test. Stratified analyses for disease activity and persistence were performed using the Pearson’s chi-squared test in different subgroups according to: the presence/absence of RF and/or ACPAs, previous exposure to bDMARDs or JAKi, and combination treatment with csDMARDs or BAR monotherapy. Kaplan–Meier curves were plotted for the evaluation of BAR persistence. SPSS version 22.0 was used.

## 3. Results

### 3.1. Baseline Population Demographics and Treatments Patterns

In total, 64 patients started treatment with BAR during the inclusion period, although only 61 fulfilled the inclusion criteria and were included in the statistical analysis. A total of 15 patients initiated BAR before the prospective study started and 13 discontinued treatment until that date. Therefore, those data were collected retrospectively.

Baseline demographics and clinical characteristics are shown in Table 1. Most patients were female, 51/61 (83.6%), with a mean (SD) age at initiation of BAR of 58.1 (15.4) years and a mean RA disease duration of 13.9 (8.3) years. In total, 48 (78.7%) patients were positive for RF and/or ACPA, and more than a half, 34/61 (55.7%), had erosive disease. In total, 30 (49.1%) patients were under glucocorticoid treatment when BAR treatment started. 

Regarding previous exposure to bDMARDs, 20 (32.8%) patients were naïve to bDMARDs or JAKi, and the median (25–75 IQR) number of previous bDMARDs or JAKi was 2 (0–4). Among patients with previous exposure to b/sdDMARDs (41/61), all patients had been treated with TNFi, while approximately one third of the overall population was first exposed to different non-TNF biologic targeted therapies (Table 1). A total of 7 (11.5%) patients had experienced previous failure to one JAKi, tofacitinib. Regarding combination treatment, 51 (83.6%) patients had used BAR in combination with csDMARDs. The mean follow-up time of the study population was 19.1 (1.4) months with a range of 3.1–51.4 months.

### 3.2. Effectiveness

A significant change in DAS28CRP was observed at the end of the follow-up period (difference of 1.2, *p* = 0.000) (Table 2). The median exposure to BAR was 12.4 (6.6–31.2) months.

**Figure 2:**
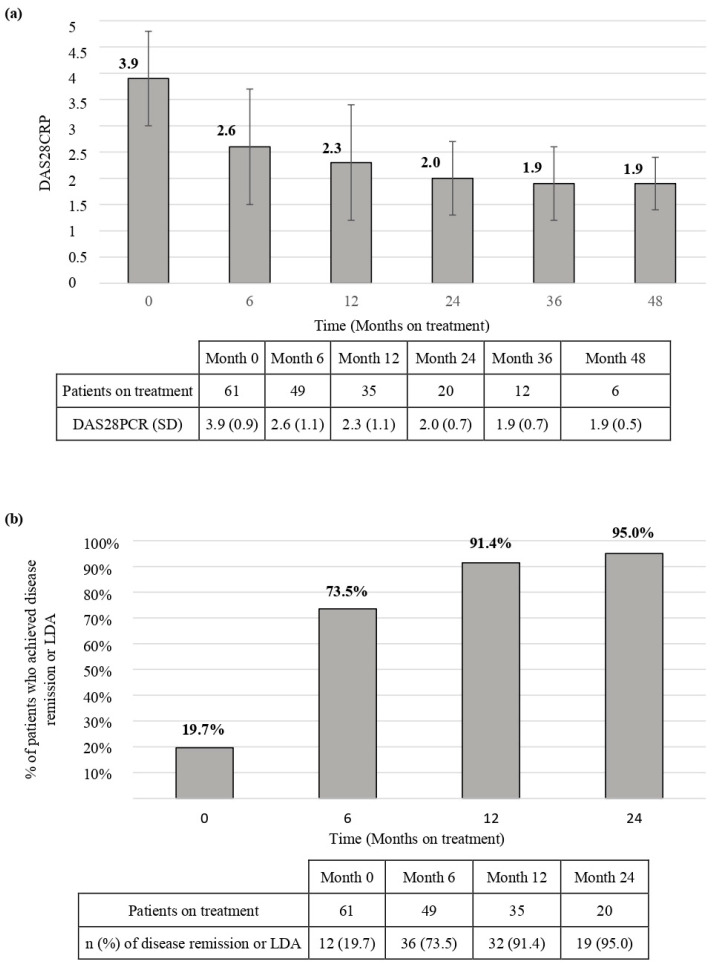
Baricitinib effectiveness. Change in disease activity from the baseline, assessed by the disease activity score using 28-joint counts-C reactive protein (**a**) and the proportion of patients who achieved disease remission or low disease activity at months 6, 12, and 24 (**b**). DAS28CRP: Disease activity score using 28-joint counts-C reactive protein; SD: standard deviation; LDA: low disease activity.

According to DAS28CRP, 37/61 (60.7%) patients achieved disease remission, whereas 43/61 (70.5%) achieved LDA along the follow-up period. The evolution of DAS28CRP along the follow-up period and the proportion of patients who achieved disease remission or LDA at months 6, 12, and 24 are shown in Figure 2.

At the end of the follow-up period, 33/61 (54.1%) patients exhibited good response, 10/61 (16.4%) moderate response, and 18/61 (29.5%) no response according to EULAR criteria.

Combined LDA/remission rates under BAR treatment were similar in patients with RF and/or ACPA positive status and in those with a negative status, (70.8% [34/48] vs. 69.2% [9/13] [*p* = 0.911]). Notably, LDA/remission rates were significantly higher in bDMARDs/JAKi-naïve patients compared to previously exposed patients, (95.0% [19/20] vs. 58.5% [24/41], respectively [*p* = 0.014]). According to the number of previous bDMARDs/JAKi, global LDA/remission rates varied from 95.0% (19/20) in bDMARDs/JAKi-naïve patients to 66.7% (4/6) in patients with one previous bDMARDs/JAKi, and 57.1% (20/35) in patients with two or more previous drugs (*p* = 0.040). Finally, no significant differences were found between patients on monotherapy with BAR and patients on combination regimen with csDMARDs, 70.0% (7/10) vs. 70.6% (36/51), respectively (*p* = 0.970).

### 3.3. Persistence

**Figure 3:**
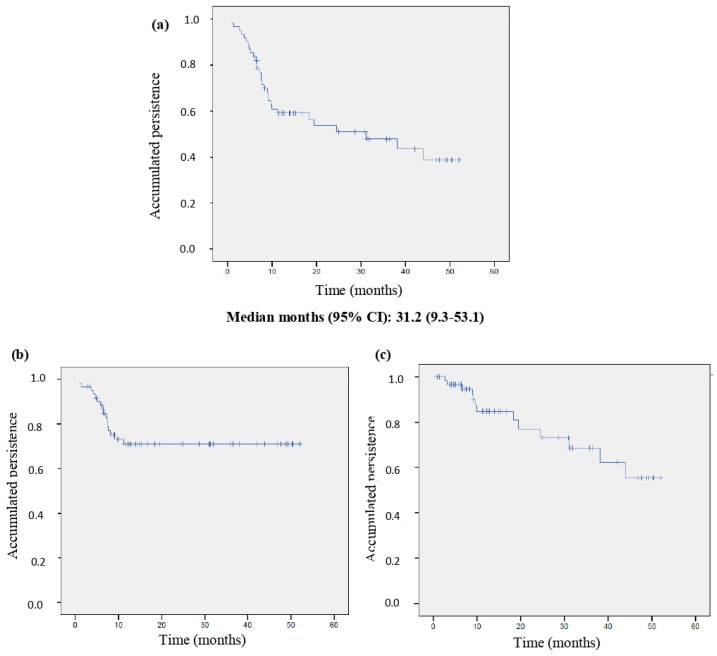
Baricitinib persistence. Drug retention in the global population (n = 61) (**a**); in patients who discontinued treatment due to loss of effectiveness (n = 16) (**b**), or due to intolerance/safety issues (n = 10) (**c**). CI: confidence interval.

In total, 31 (31/61, 50.8%) patients remained on treatment with BAR at the end of the follow-up period, with a mean time on treatment of 12.4 (6.6–31.2) months and a median persistence of 31.2 (9.3–53.1) months. The Kaplan–Meier curves for the global population and those stratified according to the cause of BAR discontinuation are shown in Figure 3.

**Figure 4:**
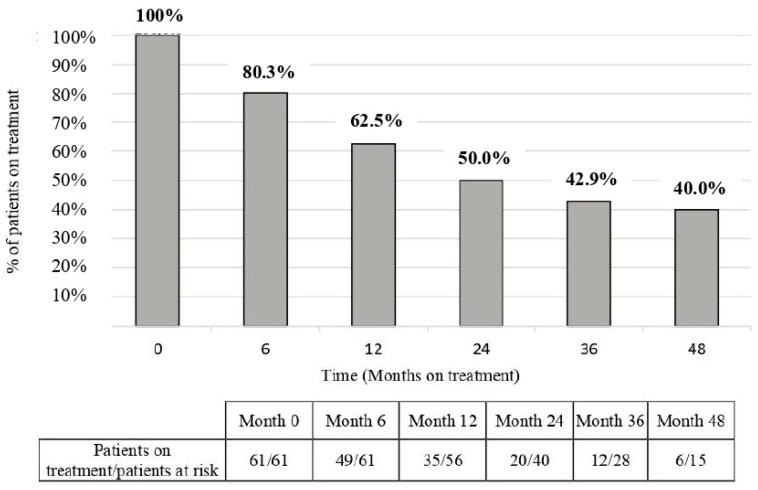
Retention rates of baricitinib treatment at different time points.

During follow-up, BAR treatment was discontinued in 16/61 (26.2%) patients due to the lack of effectiveness, and in 13/61 (21.3%) patients due to intolerance/safety issues. Finally, 1/61 (1.6%) patient ended BAR treatment by their own decision. The retention rates at different time points are shown in Figure 4.

**Figure 5:**
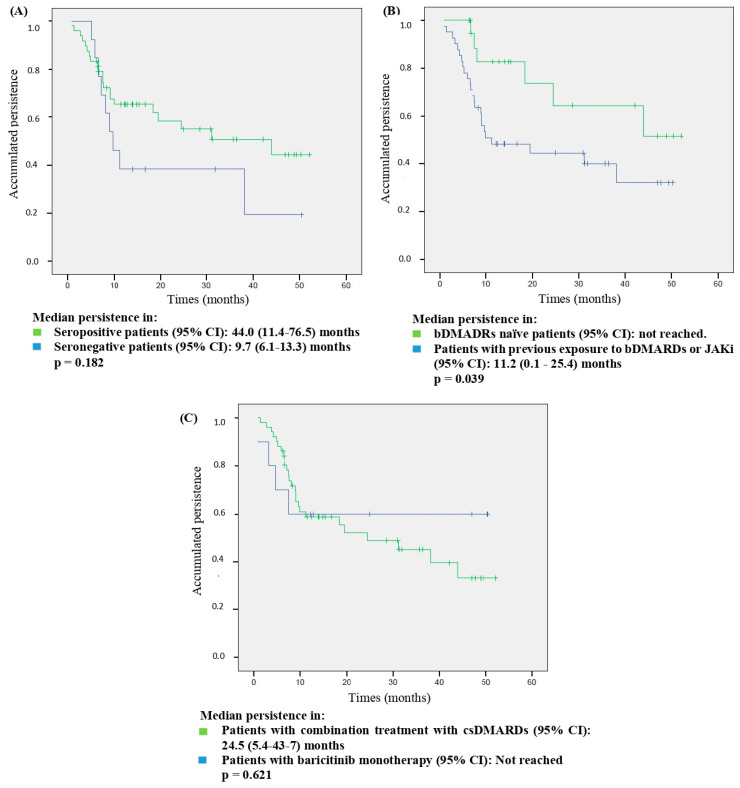
Baricitinib persistence in different subgroups according to: (**A**) the presence/absence of the rheumatoid factor and/or anti-citrullinated protein antibodies; (**B**) previous exposure to bDMARDs or JAKi; (**C**) and combination treatment with csDMARDs or baricitinib monotherapy. The log-rank test was used to compare Kaplan–Meier curves. CI: confidence interval; csDMARDs: conventional synthetic disease-modifying anti-rheumatic drugs, bDMARDs: biological disease-modifying anti-rheumatic drugs; JAKi: Janus Kinase inhibitors.

The stratified analysis of persistence according to the presence/absence of RF and/or ACPAs, previous exposure to bDMARDs or JAKi, and in combination treatment with csDMARDs or BAR monotherapy is shown in Figure 5. The log-rank test indicated a significant difference only in the stratified analysis by previous exposure to bDMARDs/JAKi: median persistence was not obtained in naïve patients (the median could not be calculated as it did not reach a 0.5 probability) vs. 11.2 (0.1–25.4) months in the group of patients with prior exposure (*p* = 0.039).

### 3.4. Adherence

The average MPR of all patients was 0.96 (0.08). According to this parameter, all patients but one were adherent to treatment. In total, 46 patients in the prospective study completed the CQR5 questionnaire, and all of them were considered “good adherents”.

### 3.5. Safety

AEs occurred in 40/61 (65.6%) patients, with an IR per 100 PY of 15.2 (95% CI 15.4–15.1), while severe AEs (SAEs) occurred in 9/61 (14.8%) patients, with an IR per 100 PY of 3.5 (3.3–3.7) (Table 3). 

The most prevalent AEs were anemia in 24/61 (39.3%) patients, infection in 22/61 (36.1%), hypercholesterolemia in 20/61 (32.8%), and abnormal liver enzymes in 19/61 (31.1%). Herpes Zoster (HZ) infection occurred in 7/61 (11.5%) patients (Table 3). Regarding laboratory parameters during BAR treatment, significant changes from the baseline values were only found in mean hemoglobin concentration (13.5 [1.5] g/dL vs. 12.9 [1.4] g/dL, *p* = 0.000) (Table 2).

A total of 13 out of 61 (21.3%) patients discontinued BAR treatment due to toxicity. Three of them discontinued treatment due to HZ infection; three due to cancer (two lung carcinoma and one breast carcinoma), two due to grade 2 anemia; two due to grade 2 abnormal liver enzymes; one patient due to grade 4 hypertriglyceridemia, grade 4 abnormal liver enzymes, and grade 3 urticaria; one patient due to increased platelet count (>1,000,000 cells/mm^3^); and one patient due to central retinal vein occlusion. No deaths were recorded.

## 4. Discussion

The main findings in our study were the high rates of effectiveness, persistence, and adherence of BAR in a long-standing and mostly bDMARD experienced population with a significant proportion of seropositivity and erosive disease; nonetheless, biologic-naïve patients achieved a better response to BAR treatment.

BAR has recently been incorporated into RA therapy after favorable efficacy results in randomized clinical trials [5,6,7,8]. In clinical practice, BAR has been postulated as one of the alternatives for unmet therapeutic needs in RA patients; in addition, recent safety concerns with tofacitinib [9] have led regulatory agencies to endorse measures to minimize risks in all JAKi treatments for chronic inflammatory diseases [13,23]. Accordingly, a repurposing of these drugs in the RA armamentarium, at least in some subpopulations, has emerged. Our safety data from a population not selected following current recommendations may provide additional information to that on published RWD on BAR treatment [4,24,25]. Unlike most real-world studies, we provide long-term safety data as adjusted incidence rates (IR) per 100 patient years, demonstrating an acceptable safety profile of BAR.

The characteristics of our patients (age around 60 years, long-standing RA with poor prognostic factors and few patients naïve to biologics or JAKi) are in line with most of the compiled real-world evidence [4,25]. In contrast, our use of BAR in monotherapy is less frequent than that of most published studies, which also show wide geographical heterogeneity [4].

Regarding effectiveness, a recent systematic review of BAR RWD reveals that most of the studies report LDA/disease remission rates after a six-month follow-up [25]. Herein we report high rates of LDA/disease remission in extended timeframes, in accordance with the results of a long-term study [26], as well as EULAR response at the end of follow-up. The rapid decrease in DAS28CRP was detected in the first six months, which led to sustained remission, i.e., more than 70% of patients in remission at 6 months of follow-up and more than 90% at 12 or successive months. These findings corroborate the effectiveness of BAR observed in other real-world studies [27,28,29,30,31,32,33,34,35,36].

BAR treatment effectiveness was not significantly affected by the presence of RF and/or ACPAs, nor by combination treatment with csDMARDs, in accordance with studies by Takahasi et al. [27] and Guidelli et al. [37], but contrary to results from Iwamoto et al. [28], in which patients on combination treatment with MTX achieved a better response. In our study, patients naïve to bDMARDs or JAKi treatment had higher LDA and disease remission rates than patients with previous exposure to bDMARDs, in line with previous studies [27,28,36,37]. Therefore, this result supports that bDMARD- naïve patients may benefit more from BAR treatment.

In our population, persistence at four years of follow-up was large; median time to BAR discontinuation was 31 months, and half of the patients remained on treatment at the end of follow-up. Retention rates at 6, 12, and 24 months were 80, 62.5, and 50%, respectively, similar to those described in other observational studies [27,29,30,31,32,36,38,39,40,41], while Japanese [42] and two Italian cohorts [43,44] showed BAR´ survival rates at 2 years higher than 70%. Beyond this time point, available RWD are limited [26,43,44]. In our study, about 40% of the refractory patients who completed the 36- or 48-month follow-up were still on treatment. In improving these results, a recent retrospective multicenter Italian study with 478 patients observed a persistence rate of 53.4% at 48 months [44]. However, compared to our population, this cohort had a lower disease duration (78 months (32–163)), lower seropositivity for RF and ACPA (60.1% and 55.2%, respectively), and fewer patients were exposed to TNFi (34%) or biologics with other mechanisms of action (1.7–17.6% for different drugs) [44]. All this data pointed to a more severe or multidrug-resistant population in our study. In this regard, a long-term extension study from Smolen et al. [26] described a lower discontinuation rate of BAR at 36 months, although in this study the patients were recruited in RCTs and were naïve to ts/b or even csDMARDs, reflecting a selected population far from the context of RWD studies. 

The Kaplan–Meier curve of persistence in patients who discontinued BAR treatment due to a lack of effectiveness showed a rapid initial decrease followed by a stabilization in the following months. This pattern indicates that most discontinuations due to lack of effectiveness were due to primary failure, as they occurred in the first months of treatment, in accordance with results of other studies [27,30,40]. Treatment discontinuations due to toxicity were more gradual over time. Our discontinuation rates related with a lack of effectiveness or adverse events match those in other studies [36,42,43,44] and are also consistent with descriptions across different JAKi [29,42,45].

The median persistence was much bigger in bDMARD- or JAKi-naïve patients than in those with prior exposure to bDMARDs or JAKi, in accordance with previous data reported in larger Spanish [31] and Italian cohorts [44] and with the conclusions of an exhaustive review of real-world studies [4]. In contrast, in the retrospective Japanese ANSWER cohort [42], the number of prior bDMARDs or JAKi did not affect JAKi (BAR and tofacitinib) retention, in accordance with other studies analyzing overall persistence of several JAKi [42,45]. However, concerning the type of prior biologic therapy, the use of IL-6Ri has been postulated as a potential risk factor for the early discontinuation of JAKi due to inefficacy [42,45]. 

In our study, statistical significance was not reached in the stratified analysis according to presence/absence of RF/ACPA, and we cannot rule out that the population size was behind these results. Indeed, the median persistence was much bigger in patients with seropositive status than in those with seronegative status (44.0 vs. 9.7 months). Interestingly, a similar trend in persistence associated with seropositive status was also described in a Spanish multicenter cohort [31], which reached statistical significance in a multicenter Italian study [37]. However, this finding was not corroborated in a recent single-center study [43] and, therefore, further research is warranted to clarify this discrepancy. Finally, no difference in persistence was found in the stratified analysis in patients with BAR monotherapy or combination treatment with csDMARDs, also in accordance with previous reports [28,31,42].

The adherence to treatment, assessed by RMP and CQR5, was high, close to 100%. These results are in contrast with a recent US experience [46] reporting only 31.8% of patients with good adherence to BAR (defined as proportion of days covered [PDC] ≥ 80%) and the poor adherence to oral RA treatment determined in a study conducted in Spain with csDMARDs [47]. In accordance with our findings, a high adherence to both JAKi (BAR and tofacitinib) was demonstrated in the study of Codes-Mendez et al. [48], suggesting that a good tolerance and rapid abrogation of symptoms can improve patient compliance with treatment. Nonetheless, due to the high adherence rates, no comparison between effectiveness and adherence was performed in our population.

Concerning safety, more than half of our patients reported AEs. However, most of them were moderate, leading to BAR discontinuation in thirteen patients and SAE occurrence in nine patients. To adjust for population size and exposure time, our safety data are shown as incidence rate per 100 PY. Comparison with similar published real-world studies is challenging as they are cohorts with limited sample sizes or shorter follow-up periods than ours and do not estimate incidence rates per PY [25]. Therefore, for SAEs we must use publications of national databases or registries, bearing in mind that our values reflect crude and not standardized IRs. In our study, the AE with the highest IR per 100 PY was anemia. In contrast to findings by Takahashi et al. [27], and Deprez et al. [38], which reported the normalization of hemoglobin values after a decrease in the first months of treatment, we observed a significant decrease in hemoglobin values along the follow-up that never led to BAR discontinuation. Regarding infections, HZ was the most common reported infection and one of the main causes of BAR discontinuation, similarly to other publications [8,27,28,49,50,51,52], although none of these infections were considered SAE. It should be noted that all but one patient with HZ were under glucocorticoid treatment at the time of infection. Indeed, glucocorticoid treatment and older age have been described as risk factors for HZ and other infections [53]. Vaccination against HZ is currently recommended for all patients prior to the initiation of JAKi treatment and could be considered for those already on treatment [54]. Only bacterial pneumonia was considered a serious infection with an IR per 100 PY of 1.1 (0.8–1.4), which ranges within the lowest SAE rates for BAR reported by Salinas et.al in a meta-analysis of multi-databases using disease registries and claims [55], although we used crude and not standardized adjusted IRs.

Regarding cardiovascular SAEs, in addition to findings with tofacitinib in the oral surveillance trial for RA [9], several RWD studies with JAKi have observed an increased risk of major adverse cardiovascular events (MACE) or thromboembolism in elderly patients with certain cardiovascular risk factors [55,56,57]. No MACE was recorded in our cohort and only one venous thrombotic event was observed in line with standardized incidence rates described by Uchida et al. [53] or in the meta-analysis published by Salinas et al. [55].

Three patients discontinued BAR due to a new diagnosis of cancer, with a crude IR per 100 PY of 1.1 (0.8–1.4), consistent with known data from RWD studies [35,50,52,53,55,58]. The two patients with lung carcinoma were ≥65 years old and smokers, two conditions in which treatment with BAR would not have been initiated following current recommendations.

To conclude, despite the severity of RA in our population, we have not found any increase in SAEs compared to the safety profile reported in RWD studies. However, given recent recommendations, it is necessary to assess inter-individual risk/benefit ratio at the initiation of BAR treatment.

This study has some limitations. First, those related to the non-interventional, ambispective design. Second, the limited sample size; we cannot rule out that a larger population could have provided significant differences in some outcomes of the stratified group analysis. Third, the single center population can limit the generalization of the results, although our findings are consistent with those reported in multicenter experiences in our country [31]. Finally, we did not collect information on the smoking status, body mass index, or other confounding variables that could interfere with the therapeutic response [59] or interpretation of safety data. 

## 5. Conclusions

In this real-world study, BAR was mainly used in patients with moderate, erosive, seropositive, long-standing RA, previously exposed to more than one b/tsDMARDs. These characteristics have been associated with more severe disease and a greater difficulty in reaching the therapeutic target. Despite this, BAR can provide significant benefits in several outcomes in RA patients, even in those with long-standing, severe, and refractory disease. However, patients without previous exposure to biologics appear to benefit more from the drug. A good adherence and the acceptable safety profile of BAR contribute to a high persistence. Together with considering safety concerns, which are mandatory in the selection of treatment candidates, all previous data support a good risk/benefit ratio of BAR in daily clinical practice. Additional prospective studies with a greater sample size are needed to confirm these findings.

## Figures and Tables

**Table 1:** Baseline characteristics of the study population (*n* = 61).

Gender (*n*, % female)	51 (83.6)
Age at initiation of BAR (years, mean, SD)	58.1 (15.4)
Disease duration (years, mean, SD)	13.9 (8.3)
RF positive (*n*, %)	47 (77.0)
ACPAs positive (*n*, %)	43 (70.5)
Erosive disease (*n*, %)	34 (55.7)
Extra-articular disease (*n*, %)	26 (42.6)
Rheumatic nodules	10 (16.4)
Sjögren syndrome	7 (11.5)
Interstitial pneumonitis	4 (6.6)
Neuropathies	2 (3.3)
Peripheral ulcerative keratitis	1 (1.6)
Raynaud syndrome	1 (1.6)
Felty syndrome	1 (1.6)
Glucocorticoid treatment (*n*, %)	30 (49.1)
Naïve to bDMARDs or JAKi treatment (*n*, %)	20 (32.8)
Previous exposure to bDMARDs or JAKi (*n*, %)	41 (67.2)
Number or previous bDMARDs	
One previous bDMARD	6 (9.8)
Two previous bDMARDs	14 (23.0)
Three previous bDMARDs	8 (13.1)
Four previous bDMARDs	7 (11.5)
Five previous bDMARDs	2 (3.3)
Six previous bDMARDs	2 (3.3)
Seven previous bDMARDs	1 (1.6)
Eight previous bDMARD	1 (1.6)
Type of previous bDMARDs	
TNFi	41 (67.2)
IL-6Ri	18 (29.1)
CTLA4-Ig	19 (31.1)
Anti-CD20 B cell depletion	21 (34.4)
Previous exposure to one JAKi (tofacitinib) (n, %)	7 (11.5)
BAR monotherapy (n, %)	10 (16.4)
BAR in combination with csDMARDs	51 (83.6)
Methotrexate	31 (50.8)
Leflunomide	14 (23.0)
Hydroxychloroquine	3 (4.9)
Sulfasalazine	2 (3.3)
Methotrexate plus leflunomide	1 (1.6)
Baseline DAS28CRP (mean, SD)	3.9 (0.9)
Baseline ESR (mml/h, mean, SD)	27.8 (23.2)
Baseline CRP (mg/dL, mean, SD)	2.0 (4.8)

ACPAs: Anti-citrullinated protein antibodies; BAR: baricitinib; bDMARDs: biologic disease-modifying anti-rheumatic drugs; csDMARDs: conventional synthetic disease-modifying anti-rheumatic drugs; DAS28CRP: Disease activity score using 28-joint counts-C reactive protein; ESR: Erythrocyte sedimentation rate; IL-6Ri: IL6 receptor inhibitors; RF: Rheumatoid factor; SD: Standard deviation; TNFi: TNF inhibitors.

**Table 2:** Variation in disease activity and laboratory parameters under baricitinib treatment.

	Baseline	Final	*p*
DAS28CRP (average, SD)	3.9 (0.9)	2.7 (1.3)	0.000
CRP (mg/dL, average, SD)	2.0 (4.8)	1.1 (1.7)	0.105
ESR (mml/h, average, SD)	29.0 (23,2)	25.7 (22.9)	0.604
Lymphocyte count (cells/mm^3^, mean, SD)	2641 (1501)	2482.6 (1505)	0.154
Neutrophil count (cells/mm^3^, mean, SD)	4198 (2126)	4157 (2132)	0.865
Hemoglobin (g/dL, mean, SD)	13.5 (1.5)	12.9 (1.4)	0.000

DAS28CRP: Disease activity score using 28-joint counts-C reactive protein; SD: Standard deviation; ESR: Erythrocyte sedimentation rate.

**Table 3:** Adverse events during baricitinib exposure.

	IP (n, %)	IR per 100 PY (95% CI)
**Patients with any AE (Total AEs = 104)**	**40/61 (65.6)**	**15.2 (15.1–15.3)**
Anemia	24/61 (39.3)	9.1 (9.0–9.2)
Any infection	22/61 (36.1)	8.4 (8.2–8.6)
Herpes Zoster	7/61 (11.5)	2.7 (2.4–3.0)
URTI	7/61 (11.5)	2.7 (2.4–3.0)
Skin and soft tissue infection	5/61 (8.2)	1.9 (1.7–2.1)
Bacterial pneumonia	3/61 (4.9)	1.1 (0.8–1.4)
Influenza A	2/61 (3.3)	0.8 (0.6–1.0)
Oral herpes simple	2/61 (3.3)	0.8 (0.6–1.0)
Hypercholesterolemia	20/61 (32.8)	7.6 (7.4–7.8)
Abnormal liver enzymes (ALT or AST)	19/61 (31.1)	7.2 (6.9–7.5)
Nausea and vomiting	4/61 (6.6)	1.5 (1.3–1.7)
Cancer	3/61 (4.9)	1.1 (0.8–1.4)
Alopecia	2/61 (3.3)	0.8 (0.6–1.0)
Skin disorders	2/61 (3.3)	0.8 (0.6–1.0)
Asthenia	2/61 (3.3)	0.8 (0.6–1.0)
Weight gain	1/61 (1.6)	0.4 (0.2–0.6)
Venous thrombotic event	1/61 (1.6)	0.4 (0.2–0.6)
Hypertriglyceridemia	1/61 (1.6)	0.4 (0.2–0.6)
Rhabdomyolysis	1/61 (1.6)	0.4 (0.2–0.6)
Platelet increase	1/61 (1.6)	0.4 (0.2–0.6)
**Patients with SAEs (Grade 3–4) (Total SAEs = 11)**	**9/61 (14.8)**	**3.5 (3.3–3.7)**
Bacterial pneumonia with intravenous treatment	3/61 (4.9)	1.1 (0.8–1.4)
Cancer	3/61 (4.9)	1.1 (0.8–1.4)
Abnormal liver enzymes (ALT or AST)	1/61 (1.6)	0.4 (0.2–0.6)
Venous thrombotic event	1/61 (1.6)	0.4 (0.2–0.6)
Hypertriglyceridemia	1/61 (1.6)	0.4 (0.2–0.6)
Skin disorders (Urticaria)	1/61 (1.6)	0.4 (0.2–0.6)
Platelet increase	1/61 (1.6)	0.4 (0.2–0.6)

IP: incidence proportion; IR: incidence rate; PY: patient-year; CI: Confidence interval; AEs: adverse events; SAEs: severe AEs; URTI: upper respiratory tract infection; ALT: alanine transaminase; AST: aspartate transaminase.

## Data Availability

The datasets generated during and/or analyzed during the current study are available from the corresponding author on reasonable request.
